# Mediterranean alien harmful algal blooms: origins and impacts

**DOI:** 10.1007/s11356-020-10383-1

**Published:** 2020-08-17

**Authors:** Christina Marampouti, Anita G. J. Buma, M. Karin de Boer

**Affiliations:** 1 Department of Ocean Ecosystems, Energy and Sustainability Research Institute Groningen, University of Groningen, Nijenborgh 7, AG 9747 Groningen, The Netherlands; 2 Bèta Science Shop, Faculty of Science and Engineering, University of Groningen, Nijenborgh 6, AG 9747 Groningen, The Netherlands

**Keywords:** Harmful algal bloom, Invasive alien species, Mediterranean Sea, Introductions, Pathways, Toxin, Syndromes

## Abstract

Harmful algal blooms (HABs) are mostly phytoplankton blooms, which have detrimental environmental and socioeconomic impacts. The Mediterranean Sea due to its enclosed nature is of special concern since it has an enormously rich native biodiversity. Though, it is also the world’s most invaded marine ecosystem and is considered at very high risk of future invasions. The aim of this review study is to explore the origins, establishment, environmental, and socioeconomic impacts of HABs caused by nonnative algal species in the Mediterranean Sea. Based on this, it is also discussed whether HABs form an increasing threat in the basin, and what could possibly be done to prevent or to minimize their impacts. The increasing rate of their introduction and the harmful impacts that they have on the environment, economy, and human health makes it important to have accurate knowledge about HABs. Anthropogenic activities and climate change are considered the main contributors of alien invasions but also the main enablers of HAB events. Mediterranean HABs are adequately studied, but there are no studies purposefully concerning invasive microalgae species in the basin. In the present study, 20 species have been identified, and an attempt has been made to collect their introduction information, as well as known or suspected impacts. Future research should be focused on data mining, current legislation updates, and monitoring of Mediterranean coastlines.

## Introduction

Harmful algal blooms (HABs) have shown an increase in frequency, intensity, and distribution in a global scale (Ben-Gharbia et al. 2016; Van Dolah 2000). These events develop mostly in coastal and sheltered areas all around the world, such as harbors, smaller bays, and coastal lagoons that can of course be attributed to the increase of monitoring programs that came with technological progress and scientific curiosity, but also with global climate change and anthropogenic impacts. These include eutrophication, habitat modification, and human-mediated introduction of no indigenous species (Kudela et al. 2015).

### What are harmful algal blooms

Photosynthetic microalgae contribute in healthy aquatic ecosystems by being the foundation of the food web, fixing carbon, and producing oxygen. However, under certain circumstances, some species have the ability to form high-biomass or toxic proliferations of cells (“blooms”). These microalgae cause harm to aquatic ecosystems, including fauna and flora, but also to humans via direct exposure to water-borne toxins or by toxic seafood consumption (Ferrante et al. 2013). Most HAB species are microalgae that belong to the classes of Bacillariophyceae (diatoms), Dinophyceae (dinoflagellates), Dictyochophyceae, Prymnesiophyceae, Raphidophyceae, and Cyanophyceae (cyanobacteria). Dinoflagellate species are the most abundant HAB species and produce the majority of the toxins in the Mediterranean Sea (Arff and Miguez 2016). HABs are naturally occurring phenomena, present in nearly all aquatic environments (freshwater, brackish, and marine). The different types of marine HABs can be distinguished by three distinctive phenomena: anoxia (due to high biomass), intoxication of marine life, and accumulation of toxins in the food chain.

The blooms of pelagic microalgae that discolor the water have traditionally been called red tides, whether they are toxigenic or not. More specifically, in the Mediterranean, some blooms can be caused by algal species that produce toxins (e.g., *Ostreopsis ovata*) where others cause nontoxic high-biomass blooms (e.g., *Alexandrium taylorii*) (Kudela et al. 2015).

### HABs from alien invasive species

**Fig. 1:**
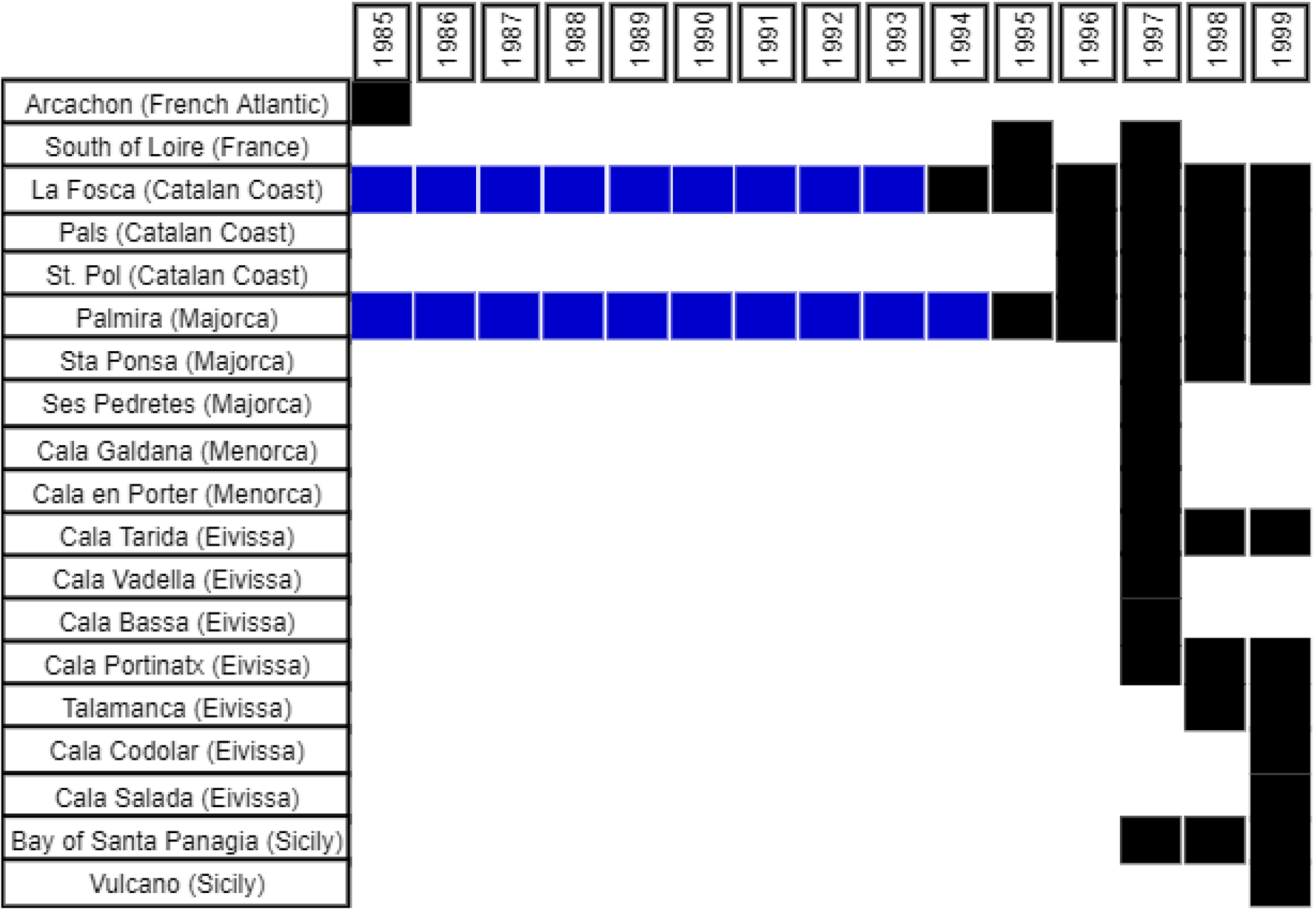
Areas of *Alexandrium taylori* blooms with up to 10^5^ cells L^−1^ in Balearic basin (Table 3 in Garcés et al. 2000)

Harmful microalgae that are found in tropical areas are expanding to temperate ecosystems probably due to water temperature rising (Ben-Gharbia et al. 2016). Evidently, HABs of pelagic and benthic microalgae are also increasing both in intensity and frequency in the Mediterranean Sea. It has been also observed that many alien microalgae blooms are co-occurring with native species blooms, but the mechanisms of their insurgence are poorly studied. Even though native Mediterranean phytoplankton species have always been manifesting blooms, alien species are a new parameter in a preexisting problem that only increases HABs in the Mediterranean waters (Streftaris and Zenetos 2006). A fine example of Mediterranean alien HAB event increase is given by Garcés et al. (2000) that reports the increase of HAB phenomena in the Mediterranean over a period of 50 years. By focusing on the findings of *A. taylori* (Fig. 1) that is considered alien, it is obvious that the species distribution is expanding, and thus, other Mediterranean areas are affected by its blooms.

**Fig. 2:**
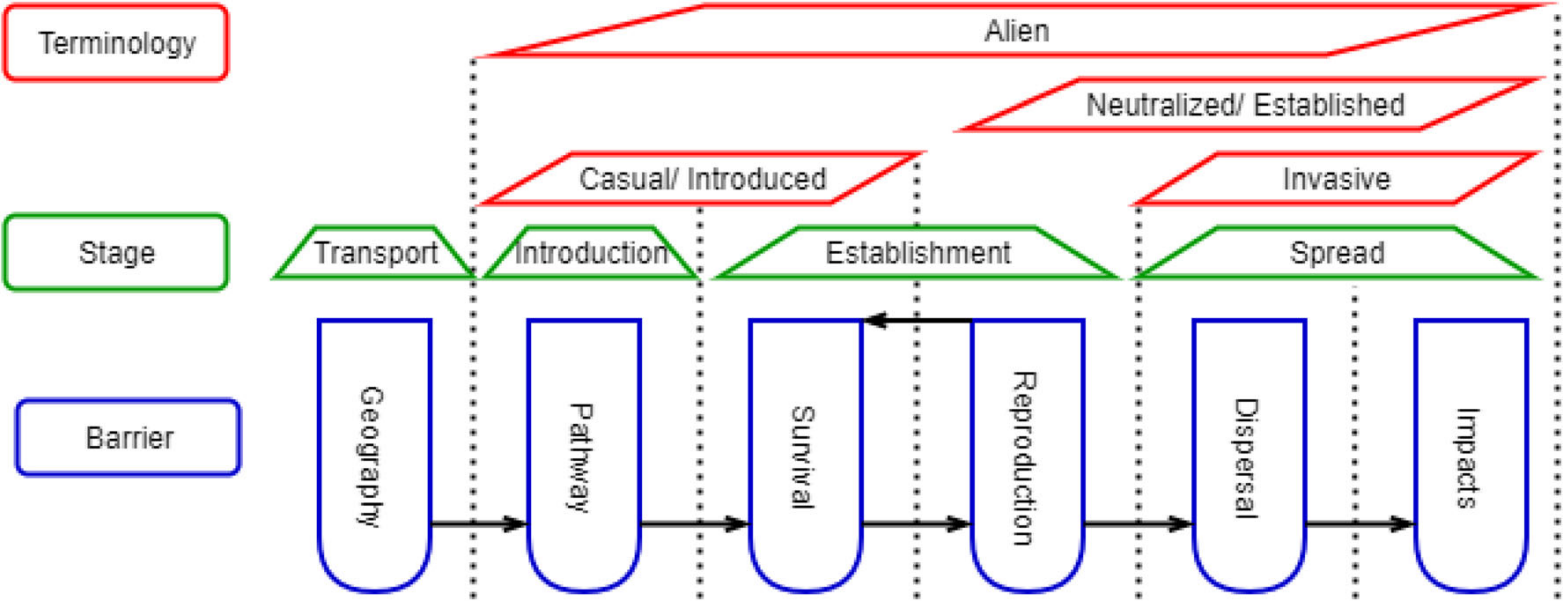
Schematic representation separating alien from invasive species (terminology). Stages of introduction of alien species (stage). The paths followed by different species to reach different stages from alien to invasive species according to their geography, pathway of introduction, survival rate, reproduction ability, further dispersal, and impacts (amendment of Fig. 1 in Otero et al. 2013)

According to the IUCN definition, agreed also by the Convention on Biological Diversity, an “invasive alien species (IAS), is an alien species which becomes established in natural or semi-natural ecosystems or habitats, is an agent of change, increases in abundance and distribution and threatens native biological diversity” (IUCN 2017). In accordance with the EU Regulation 1143/2014/EU on the prevention and management of the introduction and spread of invasive alien species, IAS are “any live specimen of a species, subspecies or lower taxon of animals, plants, fungi or microorganisms introduced outside its natural range; it includes any part, gametes, seeds, eggs or propagules of such species, as well as any hybrids, varieties or breeds that might survive and subsequently reproduce.” Following the same regulation are referred to as “an alien species whose introduction or spread has been found to threaten or adversely impact upon biodiversity and related ecosystem services.” A more precise difference in terminology between alien and invasive species is related to their stage of introduction and strength of impacts and expansion, as presented in Fig. 2.

### Mediterranean Sea

The Mediterranean Sea connects to the surrounded sea bodies via three relatively narrow pathways, the Strait of Gibraltar, the Dardanelles waterway, and the Suez Canal. Its enclosed nature makes it susceptible to alien species introduction, thus threatening the already existing rich biodiversity of the basin. The Mediterranean is considered to be the world’s most invaded marine ecosystem prone to future invasions as the anthropogenic activities and sea water temperatures are increasing (Ghabooli et al. 2013). Alien species invasions are one of the main causes for biodiversity loss in the basin because they alter the ecosystem dynamics of the marine environment in various direct but also indirect ways (Galil 2007; Coll et al. 2010; Otero et al. 2013). Their always facilitated and expanding introduction rate that extends to all Mediterranean regions poses a serious threat since they are causing unexpected and harmful impacts on the environment, economy, and also human health. They are considered “focal species”; thus, monitoring is essential in all Mediterranean regions to minimize those impacts (Katsanevakis et al. 2014a).

In the past, alterations of Mediterranean biodiversity were driven by geological and physical changes, and the Mediterranean Sea has been traditionally considered as being of low risk for harmful algal blooms due to its oligotrophic nature. However, nowadays, human activities are essential elements to consider as well, since they are one of the main reasons for habitat loss, degradation and pollution, overexploitation of marine resources, invasion of species, and of course climate change (Coll et al. 2010). The high population growth in coastal areas and the extraordinary increase in the number of harbors for recreational purposes have created suitable nutrient-rich environments for the proliferation of some algal species (Bravo et al. 2008).

Climate change also has a significant impact on coastal zones, as it drives the predicted rise in sea levels, sea, and air temperatures and also changes other hydrological characteristics of the Mediterranean coasts (Katsanevakis et al. 2014a). In the last decades, as climate change became more and more threatening, the “tropicalization” of the Mediterranean has been reported (Bianchi and Morri 2003), meaning that with the water temperature increase deriving from climate change, the temperate Mediterranean climate is becoming more and more tropic. The increased temperature of Mediterranean waters is the main reason for many alien species dispersal and establishment. There have been reported cases of species established for years in a specific Mediterranean area (resting cysts) that have not managed to expand. Although in the last years, as a result of environmental change, their population has been expanding to invasive levels (Genovesi et al. 2015; Leydet and Hellberg 2015). The increase of thermophilic biota in the Mediterranean Sea would involve changes in both indigenous (meridionalization) and non-indigenous (tropicalization) species (Saiz et al. 2014).

Before the 1980s, blooms in the Mediterranean were considered rare events (Maso and Garcés 2006). Therefore, interest from the scientific community on this topic has risen over the last decades due to their increasing distribution, from tropical waters to temperate zones such as the Mediterranean Sea (Hachani et al. 2018).

Anthropogenic activities have a direct link to Mediterranean invasions and species establishment. Pollution deriving from human pressure and climate change followed by eutrophication are known to play a key role in the increase of HAB phenomena. When nutrient loads increase, phytoplankton becomes successful over bacteria due to competition, and their growth can stimulate an algal bloom. With the current expeditiously altering Mediterranean nutrient ratios, the phytoplankton dynamics are expected to shift in the area, facilitating the expansion of non-siliceous species such as flagellates and dinoflagellates (Danovaro 2003). Consequently, together with a modification of the coastal habitats, an increase in red tide events is expected, in conjunction with the spread of toxic dinoflagellates, as already reported (Garcés et al. 2000; Vila et al. 2001). It is also safe to assume that the Mediterranean Sea is threatened from new invasions and HAB occurrences more and more (Coll et al. 2010), even though little is known about the invasion of alien phytoplankton taxa in the area (Gómez 2003). Most existing studies are focusing either on alien Mediterranean species or on HAB-causing species (native and alien) but there has not yet been a collective approach on the issue of introduced microorganisms that can cause HABs (Gómez 2010).

### Research aim

The majority of alien invasive HAB-causing species has been already identified in the Mediterranean basin; however, every year, new species are found to be present. Although mostly due to the European Union (EU) and individual countries’ initiatives, several databases and scientific papers exist that describe or contain information about these alien species. No collective database or paper exists to present only alien/invasive microalgal species that can cause HABs in the Mediterranean Sea and their impacts.

The present review study is focusing on alien/invasive species that cause HABs, their origin, vector of introduction, distribution, and impacts in the basin. The main aim of the study, apart from presenting a collective information source, is also to examine if HABs derived from introduced species are actually increasing over the last decades and why. It is hypothesized that HAB Mediterranean events are indeed increasing due to the tropicalization of the area that promotes their establishment, dispersion, and successful reproduction.

## Findings

After a thorough review of the literature, a list was composed of 20 microalgal species. From these, only 6 are referred to as invasive in the Mediterranean Sea and the remaining 14 as alien (Table 1). It is crucial to mention that more species than presented below were considered. However, not enough information could be found for those species or their invasiveness status is debatable; thus, further research is required (e.g., *Alexandrium leei*, *Alexandrium monilatum*, *Amphidoma languida*, *Tripos candelabrus*, *Prorocentrum borbonicum*, *Prorocentrum levis*, *Scrippsiella acuminate*). The final list of species consists of the most well-documented cases (e.g., *Gymnodinium catenatum* and *Ostrepsis ovata*) as well as comparably new introductions (e.g., *Prorocentrum shikokuense*).

**Table 1:** An overview of the alien/invasive Mediterranean microalgae, their invasiveness status, origin, year of introduction, vector, and their Mediterranean distribution

Species	Status	Origin	Year	Vector	Distribution
*A. andersonii*	Invasive (WoRMS 2019)	Cape cod, Massachusetts, Eastern US coast (WoRMS 2019)	1998 (Ciminiello et al. 2000)	N/A	Adriatic, Tyrrhenian Sea (Ciminiello et al. 2000)
*A. ostenfeldii*	Alien (Molnar et al. 2008; WoRMS 2019)	Iceland (WoRMS 2019)	N/A	Ballast water (Molnar et al. 2008)	Egypt, Spain (Molnar et al. 2008)
*A. pacificum*	Invasive (Genovesi et al. 2015)	Japan, Australia (Genovesi et al. 2015; Guiry and Guiry 2019)	1983 (Genovesi et al. 2015)	ballast water, shellfish aq. (Genovesi et al. 2015)	France, Italy, Spain, Western Med. (Genovesi et al. 2015)
*A. taylori*	Alien (Molnar et al. 2008; WoRMS 2019)	France, Atlantic Oc. (Guiry and Guiry 2019)	1982 (WoRMS 2019)	Ballast water, Gibraltar inflow currents (Molnar et al. 2008)	France, Central - Western Med. (Molnar et al. 2008; WoRMS 2019)
*C. bacteriastroides*	Alien (Čalić et al. 2018; WoRMS 2019)	Indian Oc. (Aligizaki et al. 2008; Čalić et al. 2018)	2010 (Čalić et al. 2018)	Levantine inflow currents (Čalić et al. 2018)	Adriatic Sea (Čalić et al. 2018)
*C. pseudosymmetricus*	Alien (Čalić et al. 2018)	Indian Oc. (Čalić et al. 2018)	2015 (Čalić et al. 2018)	Levantine inflow currents (Čalić et al. 2018)	Adriatic Sea (Čalić et al. 2018)
*C. monotis*	Invasive (WoRMS 2019)	Belgium, North Sea (WoRMS 2019)	1930s (Molnar et al. 2008)	Ballast water, oyster aq. (Molnar et al. 2008)	Widespread (Molnar et al. 2008; WoRMS 2019)
*D. acuminata*	Alien (Ignatiades and Gotsis-Skretas 2010; WoRMS 2019)	Norway, North Sea (WoRMS 2019)	N/A	N/A	Eastern Med. (Varkitzi et al. 2018)
*F. japonica*	Alien/established (Cucchiari et al. 2008)	Japan, Australia (Cucchiari et al. 2008; Gómez 2008)	1997 (Cucchiari et al. 2008)	Ballast water (Cucchiari et al. 2008)	Adriatic, Tyrrhenian Sea (Cucchiari et al. 2008)
*G. catenatum*	Invasive/cryptogenic (Zenetos et al. 2005; Katsanevakis et al. 2014a; Mozetič et al. 2017; Gladan et al. 2019)	Gulf of California, Pacific Oc. (WoRMS 2019)	1989 (Bravo et al. 1990)	Gibraltar inflow currents (Gladan et al. 2019)	Alborán Sea, South Med. (Katsanevakis et al. 2014a; Gladan et al. 2019)
*K. mikimotoi*	Alien/established/cryptogenic (Zenetos et al. 2008; WoRMS 2019)	Japan, East China Sea (Perini et al. 2018; WoRMS 2019)	1985 (AquaNIS 2015)	Ballast water (Zenetos et al. 2008)	Tyrrhenian, Aegean, Balearic Sea (Roselli et al. 2019)
*L. polyedrum*	Alien (Guiry and Guiry 2019)	Germany, North Sea (Guiry and Guiry 2019)	Before 1976 (Boni et al. 2000)	N/A	Adriatic Sea (Boni et al. 2000; Mozetič et al. 2017)
*O. ovata*	Invasive (Cohu and Lemee 2012; WoRMS 2019)	South-West Pacific Oc. (WoRMS 2019)	1979 (Gladan et al. 2019)	Shellfish aq., ballast water (Molnar et al. 2008; (Ignatiades and Gotsis-Skretas 2010)	Widespread (Streftaris and Zenetos 2006; Ghabooli et al. 2013; WoRMS 2019)
*O. siamensis*	Alien (WoRMS 2019)	Gulf of Thailand (Guiry and Guiry 2019; WoRMS 2019)	1979 (WoRMS 2019)	Ballast water (Ignatiades and Gotsis-Skretas 2010)	Widespread (Tichadou et al. 2010)
*P. emarginatum*	Alien (Zenetos et al. 2005)	Japanese Sea (Guiry and Guiry 2019; WoRMS 2019)	N/A	N/A	Greece (Ignatiades and Gotsis-Skretas 2010)
*P. shikokuense*	Alien (Roselli et al. 2019)	East China, Korean, Japanese Sea (Roselli et al. 2019)	2016 (Roselli et al. 2019)	Ballast water (Roselli et al. 2019)	South Adriatic Sea (Roselli et al. 2019)
*P. multistriata*	Invasive (Mozetič et al. 2017)	Japanese Sea (Guiry and Guiry 2019; WoRMS 2019)	1995 (Mozetič et al. 2017)	Ballast water (Mozetič et al. 2017)	Adriatic, Greece (Mozetič et al. 2017; Roselli et al. 2019)
*S. canaliculata*	Alien (Aligizaki et al. 2008)	Indo-Pacific Oc. (Aligizaki et al. 2008)	2004 (Aligizaki et al. 2008)	N/A	South-East Med. (Aligizaki et al. 2008)
*S. tropicum*	Alien (Mozetič et al. 2017; WoRMS 2019)	Tropics (Mozetič et al. 2017)	2002 (Mozetič et al. 2017)	N/A	Adriatic Sea (Mozetič et al. 2017)
*Gambierdiscus* sp.	Alien (Aligizaki et al. 2008)	Indo-Pacific Oc. (Aligizaki et al. 2008)	2003 (Aligizaki et al. 2008)	N/A	South-East Med. (Aligizaki et al. 2008)

### Final list of introduced harmful algal species

1. *Alexandrium andersonii*
2. *Alexandrium ostenfeldii*
3. *Alexandrium pacificum*
4. *Alexandrium taylori*
5. *Chaetoceros bacteriastroides*
6. *Chaetoceros pseudosymmetricus*
7. *Coolia monotis*
8. *Dinophysis acuminata*
9. *Fibrocapsa japonica*
10. *Gymnodinium catenatum*
11. *Karenia mikimotoi*
12. *Lingulodinium polyedrum*
13. *Ostreopsis ovata*
14. *Ostreopsis siamensis*
15. *Prorocentrum emarginatum*
16. *Prorocentrum shikokuense*
17. *Pseudo-nitzschia multistriata*
18. *Sinophysis canaliculata*
19. *Skeletonema tropicum*
20. *Gambierdiscus sp.*

### Distribution and origin

Even though invasive alien species origins and distribution are relatively well documented since they pose an increasing Mediterranean problem, alien microalgae are a different category due to their small size and multiple genera. They are often misidentified, like in the case of *Alexandrium pacificum* that was considered *Alexandrium catenella* in the Mediterranean until 2005 because they are both included in the *Alexandrium tamarense* species complex (John et al. 2014). Data published before 2005 which may refer to *A. catenella* could also offer information on *A. pacificum* too. It is also important to mention the absence of *Alexandrium minutum* from the list. This is because although its blooms are thoroughly studied, its origin is uncertain since it is believed to originate from Alexandria, Egypt (WoRMS 2019), thus being native to the Mediterranean. However, it is also referred to as alien with an uncertain invasiveness status in some other cases (GISD 2019). Moreover, the origin of the Mediterranean *A. pacificum* strain has been debated to be derived from Australian (Guiry and Guiry 2019) or Japanese (Genovesi et al. 2015) populations. Another species that was previously misidentified is *Karenia mikimotoi* that was confused with the nontoxic *Gyrodinium aureolum* and *Coolia monotis* that was first described in the Mediterranean as *Glenodinium monotis* (Gómez 2008). Another challenging factor was the status identification of *Gyrodynium catenatum*, since it is considered to be invasive (Katsanevakis et al. 2014b) but also cryptogenic (Zenetos et al. 2005; Perini et al. 2018). Furthermore, *Gambierdiscus* sp. is not identified to a species level in the Mediterranean (Aligizaki et al. 2008; Zenetos 2010) but some papers describing the impacts of the species are comparing it with *Gambierdiscus toxicus* (Vila et al. 2001; Aligizaki et al. 2008). Finally, *Chaetoseros bacteriastroides* and *Chaetoseros pseudosymmetricus* findings in Adriatic Sea represent the northernmost records in world’s oceans and seas. For *C. pseudosymmetricus*, this is also the first occurrence in European seas (Čalić et al. 2018). Recent records of these unusual *Chaetoceros* species in the Mediterranean Sea should be considered as an example of the expansion of thermophilic phytoplankton species.

### Toxins of alien/invasive species

It is imperative to know what kind of toxins the alien or invasive alien microalgae are co-introducing in the Mediterranean Sea and what syndromes these toxins can cause (Table 2). Toxins produced by HABs are associated with several syndromes, including paralytic (PSP), diarrhetic (DSP), amnesic (ASP), neurotoxic (NSP), and ciguatera (shell) fish poisoning (CFP), caused by consumption of contaminated seafood. Toxins are bioaccumulated by organisms that ingest algae, and thus transmitted through the food web up to humans (biomagnification).

**Table 2:** An overview of the alien/invasive Mediterranean microalgae, the abbreviation of toxins they produce, and the syndromes they may cause in the Mediterranean Sea

Species	Toxins	Syndrome
*A. andersonii*	STX, NSTX	PSP
*A. ostenfeldii*	GTX, SP	PSP, mussel toxicity
*A. pacificum*	STX, SP, goniodomins, gymnodimines	PSP
*A. taylori*	Nontoxic	N/A
*C. bacteriastroides*	Nontoxic	N/A
*C. pseudosymmetricus*	Nontoxic	N/A
*C. monotis*	Cooliatoxin	PSP
*D. acuminata*	OA, DTX	DSP
*F. japonica*	hemolytic compounds, brevetoxins	Fish killings
*G. catenatum*	STX, GTX	PSP
*K. mikimotoi*	1-acyl-3-digalactosyl glycerol, octadeca-pentaenoic acid	Fish and invertebrate mortality
*L. polyedrum*	STX, YTX,	PSP, YTX no proven effect on humans
*O. ovata*	PLTX, OVTX, ciguatoxin-like toxins, ostreocins	respiratory
*O. siamensis*	PLTX, OVTX, YTX, ciguatoxin-like toxins, ostreocin D	Clupeotoxism, respiratory
*P. emarginatum*	Unknown	Potentially, DSP
*P. shikokuense*	OA, DTX	DSP
*P. multistriata*	DA	ASP
*S. canaliculata*	N/A	N/A
*S. tropicum*	N/A	N/A
*Gambierdiscus* sp.	N/A	CFP

Paralytic (*PSP*), diarrhetic (*DSP*), amnesic (*ASP*), and ciguatera (shell) fish poisoning (*CFP*)

Some of the toxins produced by microalgae include polytoxins (PLTXs), okadaic acid (OA), dinophysistoxins (DTXs), pectenotoxins (PTXs), yessotoxins (YTXs), azaspiracids (AZAs), saxitoxins (STXs), neosaxitoxins (NSTXs), goniautoxins (GTXs), spirolides (SPs), ovatoxins (OVTXs), cooliatoxins, and domoic acid (DA) (Table 2).

One of the most toxic alien microalgae in the Mediterranean is *O. ovata* that can produce putative PLTXs, ovatoxin-a, DSP-like, and ciguatoxin-like toxins and has been shown to affect Mediterranean coastal areas. The high cell concentration of *O. ovata* that is present in sea water can affect human health in two ways; a direct contact with affected water can cause skin and respiratory disorders and in an indirect way by bioaccumulation and/or biomagnification of the produced toxins. Most human health incidents by *O. ovata* are via filter-feeding organisms (mussels), making seafood consumption alarming. More specifically, between 2006 and 2009, nine *O. ovata* blooms were observed along the French Mediterranean coastline. Five of those events affected not only tourists but also coastline inhabitants. Symptoms presented in 47 cases varied up to mild irritations of the skin, mucous membrane and the respiratory system, and regressed without any treatment after 12–72 h. In addition, five recreational beaches were also closed to the public for a short period of time (Tichadou et al. 2010).

On the other hand, the toxic diatom’s *C. monotis* Mediterranean strains does not seem to be toxic. However, further studies are needed to identify the toxicity of all alien Mediterranean algae and their effects on human health and environment. These are several irregularities and lack of knowledge; for instance, little is known about the effects of YTXs on humans (Zingone et al. 2006), and even though *P. shikokuense*’s blooms are considered toxic in Japan, no harmful effects were observed during Mediterranean blooms (Roselli et al. 2019).

### Alien invasive HAB Mediterranean events

Mediterranean HAB events are mostly related to locations with limited water exchange like harbors (Vila et al. 2001), sheltered beaches, smaller bays, and coastal lagoons (Giacobbe et al. 2000). In these enclosed areas, during summer and early autumn months, the terrestrial water inputs are limited, and thus due to solar radiation subsequently evaporation, sea levels have a tendency to decrease; thus, nutrient and salinity levels are periodically increasing boosting phytoplankton concentrations and generating blooms (Armi et al. 2012). Furthermore, biological invasions are an indication of global change. These invasions are considered to be one of the major drivers of change for biodiversity. For the Mediterranean area, HAB events of alien species induce severe environmental and economic impacts affiliated with biodiversity loss, unbalanced ecosystem, and degradation of ecosystem services such as fisheries, aquaculture, and tourism (Roselli et al. 2019). The Harmful Algal Information System (HAIS) is the main initiative that focusses on accumulating data on HAB events. They provide information on harmful algal events on HAEDAT, a database that assembles most harmful algal monitoring and management systems worldwide. With the use of the current taxonomic names of harmful algae, HAEDAT also provides biogeographical and toxicological information.

In the case of the Mediterranean, most blooms seem to form in the Western part of the basin (Table 3). This could be attributed to the focus of studies in these areas and to existing monitoring stations. For the purpose of this study, a recent update of all HAEDAT HAB-recorded events of the listed alien/invasive microalgae that occur in the Mediterranean was made (Table 3). This table gives an example of the frequency of the occurrence and distribution of these microalgae, even if limited.

**Table 3:** An overview of all HAB events in the Mediterranean until 2019 caused by the listed alien/invasive microalgae that are presented in HAEDAT (2019)

Species	Year	Location
*A. taylori*	1994; 1995; 1996; 1999 (× 3); 2000; 2001; 2004	Catalonia, Spain
	1996; 1997; 1998; 1999 (× 2); 2001; 2003 (× 2); 2004 (× 2)	Balearic Islands, Spain
*G. catenatum*	1987; 1989; 1999; 2012; 2013 (× 3); 2014; 2016	Andalucía, Spain
	1990	Valencia, Spain
	2000 (× 2); 2001 (× 2); 2002; 2003 (× 2); 2004 (× 2); 2006; 2010; 2012; 2017; 2018;	Alboran sea, Spain
	2011 (× 2)	Cadiz-Malaga, Spain
*L. polyedrum*	2009 (× 3); 2010 (× 2); 2004 (× 3); 2005 (× 2); 2009 (× 5); 2010 (× 10); 2014 (× 8); 2015 (× 4); 2016	Slovenia
	2006 (× 2)	Catalonia, Spain
*O. ovata*	2002; 2005	Ligurian Sea, Italy
	2010 (× 2); 2014; 2015; 2016; 2017; 2018 (× 2)	Catalonia, Spain
*O. siamensis*	2004; 2006	Catalonia, Spain

By taking as an example *O. ovata*, a study by Cohu and Lemee (2012) determined that different ecological factors could influence the abundances of the species. The temperature seems to have a severe influence, especially in *Ostreopsis spp.* that reported growth between 22 and 30 °C in temperate areas. Concerning light, *O. ovata* was abundant at very shallow depths showing a good adaptation to high intensity. Since the species is also considered one of the most invasive in the Mediterranean, it is useful to have an overview of its distribution to be able to better comprehend the range of alien species adjustment capability and dispersal.

Gladan et al. (2019) also presents an overview of temporal and spatial distribution of *Ostreopsis* sp. (*O. siamensis* included) events in the Mediterranean from 1972 to 2016, providing another fine example of Mediterranean alien HAB occurrence in the basin. In this study, it is also mentioned that the highest abundances of *Ostreopsis* sp. in the Mediterranean were after 2005 and also co-occurring during the negative phase of the North Atlantic Oscillation (NAO) index. This phase is resulting in fewer and weaker winter storms that bring moist air into the Mediterranean and has been related to changes in phytoplankton biomass, primary production, and toxic algal blooms.

### Introduction vectors

The Mediterranean Sea is a semi-rigid basin connected via navigational canals through the Suez Canal (since its opening in 1869) with the Red Sea, particularly in the Levant area, the Dardanelles waterway with the Sea of Marmara and coincidentally connects to the Black Sea, and also the Strait of Gibraltar with the Atlantic Ocean. Man-made canals such as the Suez Canal are the most potent mechanisms and corridors for invasions by marine species known in the world (Table 1) (Galil et al. 2016). Molecular methods demonstrate high levels of gene flow between the Red Sea, the Sea of Marmara (Black Sea), the Atlantic Ocean, and the Mediterranean populations (Otero et al. 2013).

Shipping and generally invasions by transport vector has always been an introduction vector for invasive species, even since the thirteenth century via Viking ships (Lasota et al. 2016). More specifically, ships can transport microalgal alien species in ballast water (Otero et al. 2013; Katsanevakis et al. 2014a).

Another important vector of introduction is via aquaculture. The increasing market-driven demands for exotic fish and shellfish and the decline in wild fisheries have created the need of introduced species for aquaculture. Alien microalgae are usually co-introduced with imported shellfish, or a combination of vectors such as *O. ovata* that was first introduced from the Pacific to the Atlantic Ocean through shellfish aquaculture and then into the Mediterranean through the Strait of Gibraltar (Di Cioccio et al. 2014).

In the last decades, the expansion of warm water species has been speeded up by the increase of water temperature in the Mediterranean Sea that enhances the survival of newcomers (Parravicini et al. 2015), facilitating their establishment in certain areas and under favorable conditions their expansion and invasive behavior (Čalić et al. 2018).

## Impacts

Since HABs in the Mediterranean are occurring more often and also intensifying, the risk on marine ecosystems and socioeconomy (ecosystem services) is higher. This is either due to the toxicity of the blooms (toxic HABs), or due to their ability to cause anoxic environmental conditions (high-biomass HABs). Most microalgal toxins can bioaccumulate in bivalves (filter-feeding) or other marine organisms and can affect via biomagnification not only humans but also wildlife. In many cases, these toxins can cause acute illness or death. HAB events can deem seafood consumption unsafe and decrease water quality in their occurrence range causing acute and severe, or prolonged and chronic impacts (Kudela et al. 2015). The recent update of the Mediterranean distribution, frequency and intensity of HAB species and events suggests an increase since the last decades (Table 3, HAEDAT 2019), making their impacts more extensive throughout the basin. Another possibility is that the suggested increase can be due to an increasing awareness and monitoring effort in the Mediterranean.

Because of the microalgal strain variety, it is challenging, on occasion, to determine which species are causing or could cause toxic HABs in the Mediterranean Sea. For example, *C. monotis* strains from the Mediterranean Sea have been shown not to produce toxins (Aligizaki and Nikolaidis 2008; Aligizaki et al. 2009), unlike strains from Australia that have been found to produce cooliatoxin (Amany 2014). *P. shikokuense* blooms fall into a similar category, since the Mediterranean blooms did not have any harmful effects, but they are known to be toxic in Japanese waters (Roselli et al. 2019). Furthermore, previously nontoxic strains have now been detected as toxic, like in the case of the Mediterranean strain of *A. andersonii* that has been found in Italy (Gulf of Naples) to produce a new PSP toxin and is raising concern about possible toxic events (Streftaris and Zenetos 2006).

### Socioeconomy

#### Human health

The toxins produced by marine HAB species affect human health through seafood consumption or by exposure to aerosolized toxins. In general, acute intoxication impacts of algal toxins are studied to a greater extent and are better documented than the impacts that derive from chronic environmental exposure to lower concentrations of the released toxins. Since chronic exposure impacts are poorly studied, they remain an arising issue (Ferrante et al. 2013).

Some of the health symptoms of the different syndromes are gastrointestinal disorders like nausea, diarrhea and vomiting, and muscular and neurological conditions. Numbness of mouth and extremities can last for months in the case of CFP. PSP intoxication can manifest paralysis of chest muscles and the abdominal area that can also lead to death. Symptoms such as dizziness, headaches, disorientation, confusion, short-term memory loss, and motor deficiency are attributed to ASP. Most of these syndromes are well-documented, but with scientific progress, more issues are arising such as azaspiracid shellfish poisoning (AZP) that was first diagnosed in 1990s (Kudela et al. 2015). The most common symptom observed in the Mediterranean area derives from algal aerosolized toxins and causes respiratory irritation (Table 2). More specifically, in Genoa, Italy, in 2005, during *O. ovata* and *O. siemensis* blooms, 200 people exhibited exhalatory problems after coming into contact with contaminated sea spray (Tichadou et al. 2010).

#### Aquaculture

Toxic HAB species are on the rise in many parts of the Mediterranean Sea. The produced toxins have severe effects on aquaculture because commercially important species can accumulate high levels of toxins, becoming a worldwide food safety concern for humans, in some cases causing native species mortality and also leading to farm closure (Kacem et al. 2015). For example, in the case of *G. catenatum*, the toxins are released when *G. catenatum* cells are eaten by shellfish, such as oysters, mussels, and scallops, making them poisonous to consume (ISSG 2015).

Over the past 25 years, mussels farmed along the Mediterranean coasts, mostly in the North Adriatic, have been contaminated by harmful toxins, resulting in farm closure and severe economic losses. In most cases, marine lipophilic toxins (MLTs) were the source of mussel contamination. In 1995, the DSP-related yessotoxins (YTXs) became the main lipophilic biotoxins in Adriatic shellfish (Perini et al. 2018). Most recently, in Northern Greece, at Thermaikos Gulf, the species *D. acuminata* was mostly responsible for the DSP-related intoxications of *Mytilus galloprovincialis* and long harvest closures of mussel farms over the last 15 years (Koukaras and Nikolaidis 2004; Varkitzi et al. 2018). On the other hand, nontoxic HABs caused by species like *C. bacteriastroides*, in high biomass, bigger individuals with thicker spines, can damage other organisms’ gills (Sunesen et al. 2008), thus killing native species or causing problems in aquacultured fish. Dinoflagellate blooms of *O. ovata* and *C. monotis* have also had repercussions on the economy by negatively impacting fisheries. During *O. ovata* blooms, shellfish and *Arbacia* sp. mortality has been detected on Marina di Massa reefs, and further studies have mentions of DSP-like and ciguatoxin-like toxin’s presence (Streftaris and Zenetos 2006).

#### Recreation

Some HAB events can cause water discoloration that negatively can affect the esthetic value of coastlines of areas that are economically depending on tourism and recreation (Kudela et al. 2015). In the western Mediterranean (Balearic Island, Sicily, Catalan, and Italian west coast), an increase on the dinoflagellate *A. taylori* blooms has been detected over a period of 15 years in the early 2000s. Even if the occurring blooms are nontoxic, economic losses have been reported for the impacted regions. The occurrence of high-biomass HABs during summer months can prove economically catastrophic since the deterioration of water quality damages severely the touristic industry. Similar bloom events with water discoloration and water deterioration that have been observed in the Eastern part of the Mediterranean in 2004 are also alarming (Streftaris and Zenetos 2006). A study performed by Oliveri Conti et al. (2011), focusing on *O. ovata* blooms in the Italian coastline of Ionian Sea, states that there is no imminent threat towards coastline residents, recreation, or fisheries deriving from the presence of HABs in the reported study area. However, the presence of toxic HAB species detected could be of potential future concern. On the other hand, in a later study by Funari et al. (2015), guidelines are being suggested in Italy, to minimize *O. ovata* impacts concerning recreational activities. Accidental water digestion, consumption of contaminated food, and inhalation of aerosolized toxins are the main dangers that a person can face during recreational activities. The need to put guidelines in place to facilitate recreational activities is a strong indication of an already existing and increasingly threatening problem in the Mediterranean.

### Environment

Especially the enclosed coastal areas are becoming more and more afflicted by the increase of harmful phytoplankton species. Eutrophication as well as the intentional or unintentional alteration of water circulation dynamics (dredging activities, navigational channels), the excessive use of coastal zones, and the facilitation for alien species distribution via ship ballast water contribute to the presence, frequency, and intensity of HABs making Mediterranean coastal ecosystems extremely susceptible to degradation (Armi et al. 2012). High-biomass HABs can cause ecosystem damage due to suffocation of the marine organisms via gill damage (*C. bacteriastroides*) or anoxic environmental conditions “dead zones” (*A. taylori*). Marine organism mortality can also be caused by toxin-producing HAB species (Kudela et al. 2015). For instance, the toxins produced by *G. catenatum* and *Alexandrium* spp. do not only accumulate in bivalves but also affect the bivalves’ shell movement and feeding behaviors (Bricelj et al. 1996; Escobedo-Lozano et al. 2012). *G. catenatum* toxins that have been studied along the Iberian Peninsula during blooms are various fish species. Sardines and horse mackerels, planktivorous, and zooplanktivorous fish, respectively, that are eaten by top predators have been shown to transfer biotoxins up the food chain, affecting negatively an entire ecosystem (Costa et al. 2010). Next to bioaccumulation and biomagnification of toxins, also, the high biomass of *G. catenatum* toxic blooms have been shown to affect other marine organisms via negatively affecting their environmental living conditions (Katsanevakis et al. 2014a).

## Discussion

### Strength of evidence

Identifying and describing the origin and impacts of alien phytoplankton species in the Mediterranean Sea have been proven challenging. Although there is vast literature of alien phytoplankton species in European seas (Gómez 2008 and references therein; databases such as AquaNIS, DAISIE, and EASIN), Gómez (2008) suggested that there is usually no supporting research with valid data about alien species behind this literature. This is due to a decrease in taxonomic expertise, under investigation, under sampling, or an insignificant dispersal of species. All the above components contributed in the exceptionally low number of confirmed invasions concerning phytoplankton species in the Mediterranean Sea (Mozetič et al. 2017).

In an always expanding world, it is difficult to pinpoint all new invasions. Especially for microalgal species, it is difficult to identify their origin and vector of introduction because their taxonomic differences are not individually undetectable with a naked eye. Climate change and anthropogenic activities contribute to the fast alteration of species status from introduced to alien, to established, to invasive. Some scientists also use the term cryptogenic to attribute a status to a species that means that there are no sufficient data or knowledge to indicate whether this species is native or alien in a specific area. With biological invasions developing into a rising hazard for biodiversity and socioeconomy, more parties are getting interested in cataloging the issue. Thus, there are a lot of existing databases and scientific papers on international and European level concerning the Mediterranean invasions. Their presence can be very useful in order to organize information about the species involved; however, these databases tend to be incomplete and not up to date since bioinvasions are such a rapidly progressing problem. Microalgae are often excluded or lacking information in comparison with other taxa because they are hard to observe and to identify. Identifying a species’ way of introduction is a highly difficult task due to the fact that some species have multiple possible ways of introduction, and the identification of the transport vector is considered to be based on the scientific knowledge of the researcher. Impact assessments are usually based on unclear data such as distribution of alien species. For species that can cause HABs, it is probably easier to identify their toxins and impacts in both socioeconomy and biodiversity. However, the severity of the impacts is up for debate for species of which their Mediterranean distribution is uncertain or lacking information. This problem exists because the more area a species occupies, the more its impact range is expanding (Katsanevakis and Moustakas 2018).

It is important to mention that this list is not complete concerning all monitoring efforts that have been done but were not published in journals or reports. The list only describes some of the most researched alien HAB-causing species in the Mediterranean Sea, and it demands further study to determine the missing information, which goes beyond the scope of this thesis.

### Ways of establishment

Biological invasions are enabled the last decades due to the rapid globalization and the always increasing human pressure that derives from shipping trade, traveling, and transport (Katsanevakis et al. 2014a). Due to several key characteristics, alien algal species can colonize new environments more successfully than other organisms. Their ability to tolerate variations of environmental conditions, the lack of natural predators in their newly introduced environment in combination with being r-strategist, and opportunistic feeding patterns constitutes them challenging to regulate (Otero et al. 2013). For example, the extensive human pressure from fish and mussel (*Mytilus galloprovincialis*) aquaculture, overfishing, and inadequate sewage waste disposal that takes place in the Greek coastal areas can facilitate not only alien species establishment but also extensive bloom formation (Varkitzi et al. 2018). In general, in the Mediterranean basin, the presence of tropical alien species with high bloom formation can lead to the tropicalization of the basin, by altering its natural dynamics, especially in the southern parts (Coll et al. 2010).

### What about the future?

Recognizing, assessing, and measuring the seascape-wide impacts of alien phytoplankton organisms are essential for successfully designing and implementing mitigation measures for the prevention of new invasions. The high increase of human population and activities in the last century especially in coastal and developing countries, alleviating the impact of HABs, is becoming more and more critical (Berdalet et al. 2015). The anthropogenic (exponential) need to exploit marine resources will result in acceleration of invasions and bloom formations of HAB species in the imminent future. Such needs can cause changes in temperature gradients, ocean acidification, light depletion, acute stratification, irregularities in nutrient dynamics, etc. The lack of sufficient knowledge of the processes induced by invasive alien HAB species works as a barrier that prevents predicting their future pervasiveness (Wells et al. 2015). Although forecasting the future possible impacts that derive from HAB presence is challenging under constantly altering parameters, such as species behavior and new introductions, current evidence suggests that their expanding distribution patterns and changes in algal community dynamics can distress previously unaffected areas in the Mediterranean and also in a global scale (Kudela et al. 2015).

The increase in artificial environments (ports, breakwaters, and semi-closed beaches) in highly populated coastal areas multiplied those habitats favorable for HAB species facilitating their establishment (Maso et al. 2003). The water discharges caused by anthropogenic activities in Mediterranean coastal areas could have an impact on the phytoplankton species composition, leading to an increase in the abundance of opportunistic species, like alien-introduced HAB species (Jenhani et al. 2019). Plastic pollution was suggested to also play a role in microalgal dispersal. Resting cysts of unidentified dinoflagellates and both temporary cysts and vegetative cells of *A. taylori*, *Ostreopsis* sp., and *Coolia* sp. were found on plastic debris (Maso et al. 2003). Thus, future research on the matter is suggested in order to mitigate alien microalgae dispersal in the Mediterranean. Another factor that facilitates not only microalgae establishment but also further dispersal and introduction in the basin is of course shipping. Shipping vessels are considered to have introduced one-fifth of the alien species found in the Mediterranean. The regionwide increase in shipping activities can directly be connected with biological invasions. By developing new trade patterns that can result in new shipping routes improve the water quality in port environments; managing ballast water, decreasing combining introduction vectors when is possible and cannot be avoided, and rising awareness and research possibilities, HAB Mediterranean events could be easier to monitor and mitigate (Coll et al. 2010).

### Measures to reduce risk

As biological invasions are an expanding problem, and HABs in the Mediterranean Sea are increasing in frequency and dispersal, measures are needed to be taken in order to protect the biodiversity and the neighboring countries’ populations from HAB events. Most legislations, initiatives, and projects are initiated by the EU but also from Mediterranean countries and researchers individually.

Efforts have been made worldwide to prevent and mitigate the negative effects of HABs in the last few decades. The most successful advances have been achieved through coordinated HAB monitoring and scientific research, integrated with end users and management through effective and informed policy decisions. Constant monitoring, modeling and prediction, prevention by efficiently improving coastal water quality, further research, and international coordination are the key factors in better understanding, predicting, and mitigating HABs, not only in the Mediterranean but worldwide. More specifically, measures to reduce risk could vary from observational approaches of water discoloration, to predetermined constant monitoring of smaller commercial vessel’s ballast water. More recently, several technological approaches have been made for the early detection of HABs. Satellite remote sensing, empirical, and numerical models can be put in place, but also ferry box systems, automated water sampling equipment, etc. are tools that can be equipped on smaller commercial vessels to help monitor more closely HAB occurrences, since these untreated vessels play a big role in HAB species dispersal (Anderson et al. 2017).

Unfortunately, HABs cannot easily be eliminated or prevented, but they can be monitored and predicted, and their potentially negative impacts can be managed and mitigated. Changes in human activities and behavior could also contribute to prevent or minimize certain HABs and their effects (Kudela et al. 2015).

Since HABs are a natural phenomenon, it could be assumed that there are natural ways of mitigating them. Some organisms, such as the invasive copepod *Acartia tonsa*, have been shown to be able to change energy and matter flows between pelagic and benthic compartments, modify trophic structure of invaded ecosystems, and thus even serve as a potential biological control of algal blooms (Katsanevakis et al. 2014b). *Crepidula fornicata*, an invasive Mediterranean sea snail, is another example since its feeding activities may prevent blooms of harmful algae as well (Cebrian et al. 2012).

### Current legislations

The Convention on Biological Biodiversity (CBD) understands the need for the “compilation and dissemination of information on alien species that threaten ecosystems, habitats, or species to be used in the context of any prevention, introduction and mitigation activities,” and calls for “further research on the impact of alien invasive species on biological diversity” (CBD 2000). The objective set by Aichi Biodiversity Target 9 is that “by 2020, invasive alien species and pathways are identified and prioritized, priority species are controlled or eradicated, and measures are in place to manage pathways to prevent their introduction and establishment.” This reflects Target 5 of the EU Biodiversity Strategy (EU 2011). The Marine Strategy Framework Directive (MSFD; EU 2008) identifies alien marine species as a major threat to European biodiversity and ecosystem health, compelling member states to establish strategies to achieve the “Good Marine Environmental Status” (Katsanevakis et al. 2014a). In 2004, the International Maritime Organization (IMO) released the International Convention for the Control and Management of Ship’s Ballast Water and Sediments (BWM Convention 2004). This action supervises the management of ballast waters, as they are the primal vector for the dispersion of harmful marine organisms. This regulation is regarding all potentially harmful alien species, cryptogenic but also all impacting endemic marine species and pathogens (HAOP) in the entirety of marine environments around the world. Apart from the BMW Convention, the EU Marine Strategy Framework Directive (2008/56/EC) and the EU Regulation on the prevention and management of the introduction and spread of NIS (Regulation (EU) n. 1143/2014) are also policies focusing on the oversight of endemic species (Roselli et al. 2019). The MSFD pursues an environmental perspective, by referring to the detrimental impacts that derive mostly from anthropogenic activities and result in marine water deterioration and eutrophication. A proper assessment of eutrophication involves associating data on algal species composition and biomass, but also HAB occurrence in accordance with nutrient levels availability. As a primary ecological indicator, phytoplankton is also one of the “Biological Quality Elements” (BQE) of the Water Framework Directive for the coastal water quality assessment (WFD 2000/60/EC) providing preliminary data for an area’s environmental health assessment (Varkitzi et al. 2018).

Other worth mentioning initiatives include the Mediterranean Action Plan of UNEP where 21 Mediterranean countries are collaborating to meet the challenges of protecting the marine and coastal environment while boosting regional and national plans to achieve sustainable development, and the International Society for the Study of Harmful Algae (ISSHA).

## Conclusion and recommendations

The Mediterranean Sea is undoubtedly highly impacted by alien invasions. Climate change that is causing the “tropicalization” of the Mediterranean and anthropogenic activities in coastal areas is degrading water quality, shifting temperature ranges and altering the nutrient ratio leading to ecological imbalance. These circumstances are allowing alien algal species not only to get established but also to produce high-biomass HABs. Such blooms are responsible for biodiversity and socioeconomic loss by impacting negatively marine organisms and human health.

This ecological imbalance in the Mediterranean not only provides favorable conditions for future algal introductions and bloom events but also is considered responsible for the increase of alien species. On the other hand, it is important to mention that scientific focus has shifted towards HABs in the last years because of their harmful nature, and that could also be a reason for the increase of the documentation of Mediterranean HABs.

No experimental or applied research has been found that considers all changing environmental conditions attributed to climate change that affect the increase of HABs. However, the increase of temperature in connection with the increase of HABs in every research examined is presented as a fact. Logically, that is the case, but this fact in the present review was deducted from the examined studies (e.g. Garcés et al. 2000). It is mentioned that alien-recorded HAB events seem to be increasing after 1980s (Maso and Garcés 2006). Without excluding the fact that more intense scientific research has also taken place, the other logical deduction is that since higher temperatures facilitate the expansion of alien algal species, their abundance is also increasing. Adding alien HAB events to already existing native ones is a way of facilitating on occasion their bloom cooccurrence, frequency, and density. By considering the above, the hypothesis has been verified and alien HABs are indeed an addition to an already existing situation, making it an actual problem.

For future research on changing biodiversity under changing environmental conditions, it is imperative to closely monitor alien algal species and to carry out mesocosm experiments in the field with natural water samples. Further knowledge is required in order to predict whether, where, and when changes should be expected in HAB frequency and density or reformations in community structure aiming to design efficiently mitigation and management measures. Primarily focus should be given on invasion pathway/vector management to minimize the risks of new introductions (Ojaveer et al. 2015); thus, monitoring programs should be always present in Mediterranean ports (Magaletti et al. 2018), which are common liable areas for alien and invasive species (Roselli et al. 2019).

The invasion of alien HAB species will continue to change the biodiversity of the Mediterranean Sea (Coll et al. 2010) and requires a more organized scientific approach, concerning mostly data that are scattered in various databases; update of existing data and the creation of one updated database that concerns Mediterranean alien microalgae species could prove useful in future scientific research.

## References

[CR1] Aligizaki K, Nikolaidis G (2008). Morphological identification of two tropical dinoflagellatesof the genera Gambierdiscus and Sinophysis in the Mediterranean Sea. J Biol Res-Thessaloniki.

[CR2] Aligizaki K, Katikou P, Nikolaidis G, Panou A (2008). First episode of shellfish contamination by palytoxin-like compounds from Ostreopsis species (Aegean Sea, Greece). Toxicon.

[CR3] Aligizaki K, Nikolaidis G, Katikou P, Baxevanis AD, Abatzopoulos AJ (2009). Potentially toxic epiphytic Prorocentrum (Dinophyceae) species in Greek coastal waters. Harmful Algae.

[CR4] Amany AI (2014). First record of Coolia monotis Meunier along Alexandria coast – Egypt. Egypt J Aquat Res.

[CR5] Anderson DM, Boerlage SFE, Dixon MB (Eds), Harmful algal blooms (HABs) and desalination: a guide to impacts, monitoring and management. paris, Intergovernmental Oceanographic Commission of UNESCO, 2017. 539 pp. (IOC Manuals and Guides No.78.) (English.) (IOC/2017/MG/78)

[CR6] AquaNIS. Editorial Board (2015) Information system on aquatic non-indigenous and cryptogenic species. World Wide Web electronic publication. www.corpi.ku.lt/databases/aquanis. Version 2.36+. Accessed 2019-06-30

[CR7] Arff J, Miguez BM (2016) Marine microalgae and harmful algal bloms: A European prespective. In: Tsaloglou MN (eds) Microalgae: Current research and applications. Caister Academic Press 45–71. 10.21775/9781910190272.04

[CR8] Armi Z, Trabelsi E, Turki S, Ben MN, Mahmoudi E (2012). Composition and dynamics of potentially toxic dinoflagellates in a shallow Mediterranean lagoon. Int J Oceanogr Hydrobiol.

[CR9] Ben-Gharbia H, Yahia OKD, Amzil Z, Chomérat N, Abadie E (2016). Toxicity and growth assessments of three thermophilic benthic dinoflagellates (Ostreopsis cf. ovata, *Prorocentrum lima* and *Coolia monotis*) developing in the Southern Mediterranean Basin. Toxins.

[CR10] Berdalet E, Fleming LE, Gowen R, Davidson K, Hess P (2015). Marine harmful algal blooms, human health and wellbeing: challenges and opportunities in the 21st century. J Mar Biol Assoc U K.

[CR11] Bianchi CN, Morri C (2003). Global sea warming and ‘tropicalization’ of the Mediterranean Sea: biogeographic and ecological aspects. Biogeographia.

[CR12] Boni L, Ceredi A, Guerrini F, Milandri A, Pistocchi R et al. (2000) Toxic Protoceratium reticulatum (Peridiniales, Dinophyta) in the North-Western Adriatic Sea (Italy). In: Harmful Algal Blooms 2000. Hallegraeff G, et al. (Eds) Intergovernmental Oceanographic Commission of UNESCO 2001

[CR13] Bravo I, Reguera B, Martinez A, Fraga S (1990) First report of Gymnodinium catenatum Graham on the Spanish Mediterranean coast. In: Graneli E, Sundrom B, Edler L, Anderson DM (eds). Toxic Marine Phytoplankton 449–452

[CR14] Bravo I, Vila M, Maso M, Figueroa RI, Ramilo I (2008) Alexandrium catenella and Alexandrium minutum blooms in the Mediterranean Sea: toward the identification of ecological niches. Harmful Algae 7:515–522

[CR15] Bricelj VM, Cembella AD, Laby D, Shumway SE, Cucci TL, Yasumoto T, Oshima Y, Fukuyo Y (1996). Comparative physiological and behavioral responses to PSP toxins in two bivalve mollusks, the softshell clam, Mya arenaria, and surfclam, Spisula solidissima. Harmful and toxic algal blooms.

[CR16] BWM Convention (Ballast Water Management Convention) (2004) International Convention for the Control and Management of Ships’ Ballast Water and Sediments. IMO (International Maritime Organization). http://www.imo.org/en/About/Conventions/ListOfConventions/Pages/International-Convention-forthe-Control-and-Management-of-Ships'-Ballast-Water-and-Sediments-(BWM).aspx (Accessed May 2019)

[CR17] Čalić M, Ljubimir S, Bosak S, Car A (2018). First records of two planktonic Indo-Pacific diatoms: Chaetoceros bacteriastroides and C. pseudosymmetricus in the Adriatic Sea. Oceanologia.

[CR18] CBD (Convention on Biological Diversity) (2000) Interim guiding principles. Conference of the Parties Decision V/8 Alien species that threaten ecosystems, habitats or species. http://www.cbd.int/decision/cop/default.shtml?id=7150 (Accessed June 2019)

[CR19] Cebrian E, Linares C, Marschal C, Garrabou J (2012). Exploring the effects of invasive algae on the persistence of gorgonian populations. Biol Invasions.

[CR20] Ciminiello P, Fattorusso E, Forino M, Montresor M (2000). Saxitoxin and neosaxitoxin as toxic principles of Alexandrium andersoni (Dinophyceae) from the Gulf of Naples, Italy. Toxicon.

[CR21] Cohu S, Lemee R (2012). Vertical distribution of the toxic epibenthic dinoflagellates Ostreopsis cf. ovata, Prorocentrum lima and Coolia monotis in the NW Mediterranean Sea. Cah Biol Mar.

[CR22] Coll M, Piroddi C, Steenbeek J, Kaschner K, Ben Rais Lasram F, Aguzzi J, Ballesteros E, Bianchi CN, Corbera J, Dailianis T, Danovaro R, Estrada M, Froglia C, Galil BS, Gasol JM, Gertwagen R, Gil J, Guilhaumon F, Kesner-Reyes K, Kitsos MS, Koukouras A, Lampadariou N, Laxamana E, López-Fé de la Cuadra CM, Lotze HK, Martin D, Mouillot D, Oro D, Raicevich S, Rius-Barile J, Saiz-Salinas JI, San Vicente C, Somot S, Templado J, Turon X, Vafidis D, Villanueva R, Voultsiadou E (2010). The biodiversity of the Mediterranean Sea: estimates, patterns, and threats. PLoS One.

[CR23] Costa PR, Botelho MJ, Lefebvre KA (2010). Characterization of paralytic shellfish toxins in seawater and sardines (Sardina pilchardus) during blooms of Gymnodinium catenatum. Hydrobiologia.

[CR24] Cucchiari E, Guerrini F, Penna A, Totti C, Pistocchi R (2008). Effect of salinity, temperature, organic and inorganic nutrients on growth of cultured Fibrocapsa japonica (Raphidophyceae) from the northern Adriatic Sea. Harmful Algae.

[CR25] Danovaro R (2003). Pollution threats in the Mediterranean Sea: an overview. Chem Ecol.

[CR26] Di Cioccio D, Zingone A, De Girolamo P (2014) Ecology of the toxic dinoflagellate Ostreopsis cf. ovata along the coasts of the Campania region (Tyrrhenian Sea, Mediterranean Sea). PhD Programme. Università degli Studi di Napoli “Federico II”

[CR27] Escobedo-Lozano AY, Estrada N, Ascencio F, Contreras G, Alonso-Rodriguez R (2012). Accumulation, biotransformation, histopathology and paralysis in the Pacific Calico Scallop *Argopecten ventricosus* by the paralyzing toxins of the dinoflagellate *Gymnodinium catenatum*. Mar Drugs.

[CR28] EU (2008) Directive of the European Parliament and the Council Establishing a Framework for Community Action in the Field of Marine Environmental Policy (Marine Strategy Framework Directive). European Commission. Directive 2008/56/EC, OJ L 164

[CR29] EU (2011) European Parliament resolution of 20 April 2012 on our life insurance, our natural capital: an EU biodiversity strategy to 2020 (2011/2307(INI)), COM/2011/244, European Commission, Brussels, 16 pp

[CR30] Ferrante M, Sciacca S, Fallico R, Fiore M, Conti GO (2013). Harmful algal blooms in the Mediterranean Sea: effects on human health. Open Access Sci Rep.

[CR31] Funari E, Manganelli M, Testai E (2015). *Ostreospis* cf. *ovata* blooms in coastal water: Italian guidelines to assess and manage the risk associated to bathing waters and recreational activities. Harmful Algae.

[CR32] Galil BS (2007). Loss or gain? Invasive aliens and biodiversity in the Mediterranean Sea. Mar Pollut Bull.

[CR33] Galil BS, Marchini A, Occhipinti-Ambrogi A (2016). East is east and west is west? Management of marine bioinvasions in the Mediterranean Sea. Estuar Coast Shelf Sci.

[CR34] Garcés E, Maso M, Vila M, Camp J (2000). Harmful algae events in the Mediterranean: are they increasing?. Harmful Algae News.

[CR35] Genovesi B, Berrebi P, Nagai S, Reynaud N, Wang J, Masseret E (2015). Geographic structure evidenced in the toxic dinoflagellate Alexandrium pacificum Litaker (a. catenella – group IV (Whedon & Kofoid) Balech) along Japanese and Chinese coastal waters. Mar Pollut Bull.

[CR36] Ghabooli S, Shiganova TA, Briski E, Piraino S, Fuentes V, Thibault-Botha D, Angel DL, Cristescu ME, MacIsaac HJ (2013). Invasion pathway of the ctenophore Mnemiopsis leidyi in the Mediterranean Sea. PLoS One.

[CR37] Giacobbe MG, Penna A, Ceredi A, Milandri A, Poletti R, Yang X (2000). Toxicity and ribosomal DNA of the dinoflagellate Dinophysis sacculus (Dinophyta). Phycologia.

[CR38] GISD - Global Invasive Species Database (2019) Alexandrium minutum. http://www.iucngisd.org/gisd/speciesname/Alexandrium+minutum (Accessed July 2019)

[CR39] Gladan ZN, Arapov J, Casabianca S, Penna A, Honsell G (2019). Massive occurrence of the harmful benthic dinoflagellate Ostreopsis cf. ovata in the Eastern Adriatic Sea. Toxins.

[CR40] Gómez F (2003). The toxic dinoflagellate Gymnodinium catenatum: an invader in the Mediterranean Sea. Acta Bot Croat.

[CR41] Gómez F (2008). Phytoplankton invasions: comments on the validity of categorizing the non-indigenous dinoflagellates and diatoms in European seas. Mar Pollut Bull.

[CR42] Gómez F, Briend F (2010). Changes in the Mediterranean phytoplankton community related to climate warming. Phytoplankton responses to Mediterranean environmental changes.

[CR43] Guiry MD, Guiry GM (2019). AlgaeBase.

[CR44] Hachani MA, Dhib A, Fathalli A, Ziadi B, Turki S, Aleya L (2018). Harmful epiphytic dinoflagellate assemblages on macrophytes in the Gulf of Tunis. Harmful Algae.

[CR45] HAEDAT (2019) Harmful Algae Event Database. IOC-ICES-PICES. Mediterranean. http://haedat.iode.org/index.php. (Accessed July 2019)

[CR46] Ignatiades L, Gotsis-Skretas O (2010). A review on toxic and harmful algae in Greek coastal waters (E. Mediterranean Sea). Toxins.

[CR47] IUCN (International Union for Conservation of Nature) (2017) Issues brief: invasive alien species and climate change. Downloaded from https://www.iucn.org/theme/climate-change/events/iucn-unfccc/2015-paris. Accessed July 2019

[CR48] Jenhani ABR, Fathalli A, Naceur HB, Hayouni D, Aouani J, Romdhane MS (2019). Screening for alien and harmful planktonic species in the Gulf of Gabes (Tunisia, Southeastern Mediterranean Sea). Reg Stud Mar Sci.

[CR49] John U, Litaker RW, Montresor M, Murray S, Brosnahan ML, Anderson DM (2014). Formal revision of the Alexandrium tamarense species complex (Dinophyceae) taxonomy: the introduction of five species with emphasis on molecular based (rDNA) classification. Protist.

[CR50] Kacem I, Papiol G, De la Iglesia P, Diogène J, Hajjem B (2015). Comparative toxicity and paralytic shellfish poisoning toxin profiles in the mussel Mytilus galloprovincialis and the oyster Crassostrea gigas collected from a Mediterranean lagoon in Tunisia: a food safety concern. Int J Food Prop.

[CR51] Katsanevakis S, Moustakas A (2018). Uncertainty in marine invasion science. Front Mar Sci.

[CR52] Katsanevakis S, Wallentinus I, Zenetos A, Leppäkoski E, Çinar ME (2014). Impacts of invasive alien marine species on ecosystem services and biodiversity: a pan-European review. Aquat Invasions.

[CR53] Katsanevakis S, Wallentinus I, Zenetos A, Leppäkoski E, Çinar ME (2014). Impacts of invasive alien marine species on ecosystem services and biodiversity: a pan-European review, supplementary material. Aquat Invasions.

[CR54] Koukaras K, Nikolaidis G (2004). Dinophysis blooms in Greek coastal waters (Thermaikos Gulf, NW Aegean Sea). J Plankton Res.

[CR55] Kudela RM, Berdalet E, Bernard S, Burford M, Fernand L (2015). Harmful algal blooms. A scientific summary for policy makers.

[CR56] Lasota R, Pierscieniak K, Garcia P, Simon-Bouhet B, Wolowicz M (2016). Large-scale mitochondrial COI gene sequence variability reflects the complex colonization history of the invasive soft-shell clam, Mya arenaria (L.) (Bivalvia). Estuar Coast Shelf Sci.

[CR57] Leydet KP, Hellberg ME (2015). The invasive coral Oculina patagonica has not been recently introduced to the Mediterranean from the western Atlantic. BMC Evol Biol.

[CR58] Magaletti E, Garaventa M, David M, Castriota L, Kraus R (2018). Developing and testing an early warning system for non-indigenous species and ballast water management. J Sea Res.

[CR59] Maso M, Garcés E (2006). Harmful microalgae blooms (HAB); problematic and conditions that induce them. Mar Pollut Bull.

[CR60] Maso M, Garcés E, Pages F, Camp J (2003). Drifting plastic debris as a potential vector for dispersing harmful algal bloom (HAB) species. Sci Mar.

[CR61] Molnar JL, Gamboa RL, Revenga C, Spalding MD (2008). Assessing the global threat of invasive species to marine biodiversity. Front Ecol Environ.

[CR62] Mozetič P, Cangini M, Francé J, Bastianini M, Aubry FB et al. (2017) Phytoplankton diversity in Adriatic ports: Lessons from the port baseline survey for the management of harmful algal species. Marine Pollution Bulletin 147:117-132. 10.1016/j.marpolbul.2017.12.029 10.1016/j.marpolbul.2017.12.029 29295741

[CR63] Ojaveer H, Galil BS, Campbell ML, Carlton JT, Canning-Clode J, Cook EJ, Davidson AD, Hewitt CL, Jelmert A, Marchini A, McKenzie CH, Minchin D, Occhipinti-Ambrogi A, Olenin S, Ruiz G (2015). Classification of non-indigenous species based on their impacts: considerations for application in marine management. PLoS Biol.

[CR64] Oliveri Conti G, Ledda C, Zuccarello M, Fiore M, Phallic R (2011). Detection of *Ostreopsis ovata* from Eastern Sicily Coast using SEM microscopy coupled to the Vibrio fischeri test Development. J Aquac Res.

[CR65] Otero M, Cebrian E, Francour P, Galil B, Savini D (2013). Monitoring marine invasive species in Mediterranean marine protected areas (MPAs): a strategy and practical guide for managers. Medpan North project.

[CR66] Parravicini V, Mangialajo L, Mousseau L, Peirano A, Morri C, Montefalcone M, Francour P, Kulbicki M, Bianchi CN (2015). Climate change and warm-water species at the north-western boundary of the Mediterranean Sea. Mar Ecol.

[CR67] Perini F, Bastianini M, Capellacci S, Pugliese L, DiPoi E, Cabrini M, Buratti S, Marini M, Penna A (2018). Molecular methods for cost-efficient monitoring of HAB (harmful algal bloom) dinoflagellate resting cysts. Mar Pollut Bull.

[CR68] Regulation (Eu) (2014). No 1143/2014 of the European Parliament and of the Council on the prevention and management of the introduction and spread of invasive alien species. Official Journal of the European Union.

[CR69] Roselli L, Vadrucci MR, Fanelli F, Ungaro N, Caroppo C (2019). First bloom event of the small dinoflagellate Prorocentrum shikokuense in the Mediterranean Sea: cryptogenic or introduced?. Mar Pollut Bull.

[CR70] Saiz E, Sabatés A, Gili JM, Goffredo S, Dubinsky Z (2014). The zooplankton. The Mediterranean Sea.

[CR71] Streftaris N, Zenetos A (2006) Alien marine species in the Mediterranean - the 100 ‘worst invasives’ and their impact. Mediterr Mar Sci 7(1):87–118

[CR72] Sunesen I, Hernández-Becerril DU, Sar EA (2008). Marine diatoms from Buenos Aires coastal waters (Argentina). V Species of the genus Chaetoceros. Rev Biol Mar Oceanogr.

[CR73] Tichadou L, Glaizal M, Armengaud A, Grossel H, Lemée R, Kantin R, Lasalle JL, Drouet G, Rambaud L, Malfait P, de Haro L (2010). Health impact of unicellular algae of the Ostreopsis genus blooms in the Mediterranean Sea: experience of the French Mediterranean coast surveillance network from 2006 to 2009. Clin Toxicol.

[CR74] Van Dolah FV (2000). Marine algal toxins: origins, health effects, and their increased occurrence. Environ Health Perspect.

[CR75] Varkitzi I, Markogianni V, Pantazi M, Pagou K, Pavlidou A (2018). Effect of river inputs on environmental status and potentially harmful phytoplankton in a coastal area of eastern Mediterranean (Maliakos Gulf, Greece). Mediterr Mar Sci.

[CR76] Vila M, Garcés E, Maso M (2001). Potentially toxic epiphytic dinoflagellate assemblages on macroalgae in the NW Mediterranean. Aquat Microb Ecol.

[CR77] Wells ML, Trainer VL, Smayda TJ, Karlson BSO, Trick CG, Kudela RM, Ishikawa A, Bernard S, Wulff A, Anderson DM, Cochlan WP (2015). Harmful algal blooms and climate change: learning from the past and present to forecast the future. Harmful Algae.

[CR78] WoRMS Editorial Board (2019). World Register of Marine Species. Checklist dataset 10.14284/170 accessed via GBIF.org on June 2019

[CR79] Zenetos A (2010). Trend in aliens’ species in the Mediterranean. An answer to Galil, 2009 «taking stock: inventory of alien species in the Mediterranean Sea». Biol Invasions.

[CR80] Zenetos A, Cinar M, Pancucci-Papadopoulou M, Harmelin J, Furnari G (2005). Annotated list of marine alien species in the Mediterranean with records of the worst invasive species. Mediterr Mar Sci.

[CR81] Zenetos A, Meric E, Verlaque M, Galli P, Boudouresque CF, Giangrade A (2008). Additions to the annotated list of marine alien biota in the Mediterranean with special emphasis on Foraminifera and Parasites. Mediterr Mar Sci.

[CR82] Zingone A, Siano R, D’Alelio D, Sarno D (2006). Potentially toxic and harmful microalgae from coastal waters of the Campania region (Tyrrhenian Sea, Mediterranean Sea). Harmful Algae.

